# *Leishmania amazonensis* fails to induce the release of reactive oxygen intermediates by CBA macrophages

**DOI:** 10.1111/j.1365-3024.2012.01384.x

**Published:** 2012-09-11

**Authors:** T F ALMEIDA, L C PALMA, L C MENDEZ, A A NORONHA-DUTRA, P S T VERAS

**Affiliations:** Laboratório de Patologia e Biointervenção do CPqGM-FIOCRUZ, Bahia, Brazil

**Keywords:** *Leishmania*, macrophage, reactive oxygen intermediates

## Abstract

CBA mouse macrophages effectively control *Leishmania major* infection, yet are permissive to *Leishmania amazonensis*. It has been established that some *Leishmania* species are destroyed by reactive oxygen species (ROS). However, other species of *Leishmania* exhibit resistance to ROS or even down-modulate ROS production. We hypothesized that *L. amazonensis*–infected macrophages reduce ROS production soon after parasite–cell interaction. Employing a highly sensitive analysis technique based on chemiluminescence, the production of superoxide (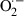) and hydrogen peroxide (H_2_O_2_) by *L. major*- or *L. amazonensis*-infected CBA macrophages were measured. *L. major* induces macrophages to release levels of 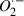 3·5 times higher than in uninfected cells. This 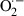 production is partially dependent on NADPH oxidase (NOX) type 2. The level of accumulated H_2_O_2_ is 20 times higher in *L. major*-than in *L. amazonensis*-infected cells. Furthermore, macrophages stimulated with *L. amazonensis* release amounts of ROS similar to uninfected cells. These findings support previous studies showing that CBA macrophages are effective in controlling *L. major* infection by a mechanism dependent on both 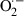 production and H_2_O_2_ generation. Furthermore, these data reinforce the notion that *L. amazonensis* survive inside CBA macrophages by reducing ROS production during the phagocytic process.

## Introduction

*Leishmania* are obligate intracellular parasites that cause either visceral or cutaneous leishmaniases. To understand the mechanisms involved in host response to *Leishmania*, studies using several mouse strains have been carried out (1). The outcome of *Leishmania* infection is determined by the early events occurring during innate immune response. The main initial events in *Leishmania*–macrophage interaction are recognition, followed by parasite internalization (2–4). Parasite recognition may induce macrophages to release reactive oxygen species (ROS), such as superoxide (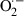). 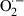 production is dependent on the recruitment of NADPH oxidase (NOX) subunits to the membrane of nascent phagosome, resulting in NOX assembly.


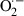
 and nitric oxide (NO) are key molecules known to be involved in the macrophage-mediated innate host defence against protozoan parasites (5–7). 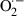
can be produced by macrophages even without any previous activation during the early contact of parasites with the host cell (5). On the other hand, NO is a molecule produced only by activated macrophages. Depending on *Leishmania* species both 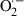 and NO play a crucial role in controlling infections (8–10). In addition to its own toxicity, 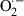 is precursor of other ROS, such as hydrogen peroxide (H_2_O_2_), hydroxyl radical (HO˙), hypochlorite (HOCl^−^) (5,6,11). These molecules can combine with NO to produce peroxynitrite (ONOO^−^) that exhibited a high toxic effect against *Leishmania* parasites (11). A recent *in vitro* study has demonstrated that there is an association between high levels of 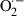 production and the significant leishmanicidal capacity of host cells (12). Nonetheless, some *Leishmania* species adopt various defence mechanisms to cope with oxidative stress, such as decrease in 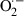 production, inhibition of NOX assembly, as well as by expression of antioxidant molecules (11,13–15).

CBA mice, while known to be resistant to *Leishmania major*, are susceptible to *Leishmania amazonensis*. This model allows the trigger mechanisms involved in *Leishmania* infection to be identified because of the static genetic background of the host (16). Additionally, CBA macrophages control *L. major* infection, while they are permissive to *L. amazonensis* infection (17). We have previously shown that interferon-gamma (IFN-γ)-stimulated CBA macrophages produce similar amounts of NO in response to *L. major* or *L. amazonensis* infection (17). However, using this model, NO produced in response to IFN-γ only played a role in controlling *L. major* infection, which suggests that *L. amazonensis* modulates or is resistant to factors that control *L. major* infection. We hypothesized that *L. amazonensis* modulates the production of microbicidal molecules other than NO, such as ROS, soon after infection, allowing parasites to survive inside CBA macrophages.

A comparative study endeavouring to evaluate the ability of macrophages to release distinct levels of ROS in response to two distinct *Leishmania* species has not been previously performed. As the 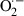 production at early stages of infection can be crucial to efficient intracellular parasite killing (12), we aimed to characterize ROS production by measuring the levels of 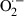 released and H_2_O_2_ generated by CBA mouse peritoneal, thioglycolate-elicited macrophages in response to *L. major* or *L. amazonensis* stimulation. The data herein show that CBA macrophages exposed to *L. major* produced high levels of ROS, yet in response to *L. amazonensis* very low levels of ROS were generated during the phagocytic process.

## Materials and Methods

### Reagents

Lucigenin (bis-*N*-methylacridinium nitrate), luminol (5-amino-2,3 dihydro-1,4-phthalazinedione sodium salt), microperoxidase, apocynin (4-hydroxy-methoxyacetophenone), Schneider’s medium, superoxide dismutase (SOD), thioglycolate and latex beads were obtained from Sigma (St. Louis, MO, USA). Dulbecco’s modified Eagle’s medium (DMEM), foetal bovine serum (FBS), L-glutamine and HEPES were purchased from Invitrogen (Carlsbad, NM, USA), and ciprofloxacin was from HalexIstar (Goiânia, Brazil).

### Parasites

*L. amazonensis* (MHOM/Br88/Ba-125) and *L. major* (MHOM/RI/−/WR-173) parasites were provided by Dr. Aldina Barral (CPqGM/FIOCRUZ). *L. major* and *L.* *amazonensis* promastigotes were maintained in Schneider’s medium plus 10% FBS for up to six passages and were expanded for 3–5 days in Schneider’s medium plus 10% FBS to reach the stationary phase, then washed with a saline solution as previously described (16) and finally adjusted to a ratio of ten parasites per macrophage (10:1 ratio).

### Thioglycolate-elicited peritoneal macrophages

All experiments were performed accordingly to the standards of the Ethics Committee on Animal Experimentation at the Oswaldo Cruz Foundation (CPqGM/FIOCRUZ). Macrophages were harvested from the 4-day thioglycolate-elicited peritoneal cavity of CBA mice as previously described (17). Briefly, macrophages were cultivated in DMEM medium at a concentration of 5 × 10^5^ cells/mL and then plated in 35-mm Petri dishes at 37°C in 5% CO_2_/95% humidified air. After 4 h, the nonadherent cells were removed and the cell cultures were incubated overnight.

### ROS production by *Leishmania*-stimulated macrophages

The ROS production by peritoneal inflammatory macrophages response to *Leishmania* stimulation was estimated using a photon-counting device monitoring chemiluminescence (CL) incorporating a gallium arsenide photomultiplier tube (Hamamatsu R943, Hamamatsu Photonics K.K., Hamamatsu City, Japan). CL emissions from sample dishes, incubated at 37°C in a sealed chamber, were reflected and focused onto the photomultiplier tube. The emitted signal was fed directly to a frequency counter unit, and data were collected in units of photon counts per second (8).

Macrophage cultures were set aside for 3 min to allow for temperature stabilization before sampling. The 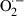 production and H_2_O_2_ formation were measured using CL. To quantify 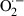 production, thioglycolate-elicited peritoneal CBA macrophages (5 × 10^5^ cells/mL) were stimulated with *L. major* or *L. amazonensis* promastigotes (10:1 ratio) during the first 30 min of parasite–host cell interaction at 37°C in the presence of lucigenin (25 μm). Macrophage cultures were maintained for 30 min at 37°C in the presence of lucigenin (25 μm) to evaluate basal 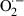 production (negative control). Opsonized zymosan particles (10:1 ratio) were used as positive (18), and latex beads (0·9 μm; 10:1 ratio) as negative controls. The rapid decay values of photon emission in response to the addition of SOD (2·5 UI/mL) were verified at the end of each assay, confirming that photon released was as a result of 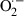 production. For H_2_O_2_ measurement, CBA macrophages were incubated with luminol (25 μm) and immediately exposed to *L. major* or *L. amazonensis* (10:1 ratio) at 37°C. After 30 min, cell supernatants were collected, and the supernatants were stored at -20°C, centrifuged at 200×*g* for 3 min prior to peroxide determination using a luminol-dependent CL assay (19). Briefly, luminol (25 μm) was added to cell supernatants, followed by microperoxidase (80 nm). The microperoxidase-dependent H_2_O_2_ decay was determined for the next 2 min.

### NOX inhibition using apocynin

Apocynin acts as an inhibitor of 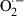 production by blocking the phosphorylation and translocation of the p47^phox^ and p67^phox^ subunits of NOX to phagosome membrane, resulting in inhibition of NOX assembly (20). To evaluate the role NOX plays in 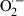 production induced by *Leishmania* spp. promastigotes, macrophage cultures (5 × 10^5^ cells/mL) were treated with apocynin (500 μm) for 18 h at 37°C and then infected with *L. major* or *L. amazonensis* promastigotes at a 10:1 ratio. 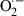 production by *L. major*- and *L. amazonensis*-infected cells treated with apocynin was measured for 10 min at 37°C in the presence of lucigenin (25 μm). Apocynin treatment (250–1000 μm) did not alter macrophage viability for 48-h culture (data not shown).

### Sequential phagocytosis assays

To test whether the parasite-induced effect on 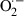 production is an active and specific *L. amazonensis*-induced mechanism, a sequential stimulation assay was used and *Leishmania*-infected macrophages were incubated with a second stimulus. Macrophages were initially incubated with *L. major* or *L. amazonensis* promastigotes for 30 min. Next, the parasite stimuli were switched, and the cells were incubated for a second 30-min period, with either *L.* *amazonensis* or *L. major* promastigotes (10:1), respectively. These sequential stimulations were performed in the presence of lucigenin (25 μm) at 37°C and 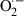 production was measured by determining photon counts emitted by stimulated cells.

### Data presentation and statistical analyses


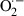
 release is represented as the average level of ROS production (*n* = 11 experiments) by inflammatory macrophages following the addition of *L.* *major* or *L. amazonensis* promastigotes. 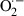 production by infected and control cells were also expressed as *R*_max_, which represents average of the highest CL response from stimulated cells. H_2_O_2_ accumulation is illustrated by a representative experiment (one of five identical experiments). The equation *R*= *R*_max_/(*T*_max_ − *T*_*i*_) was used to estimate the amount of H_2_O_2_ detected in culture supernatants of *Leishmania*-infected cells (21). *R*_max_= average of the highest CL response from stimulated cells, *T*_max_ = the point in time (seconds) at which the maximum number of photons is emitted by cells and *T*_*i*_ = the time point (seconds) at which cells begin to emit photons. All statistical tests were performed using graphpad prism 4·00 (San Diego, CA, USA), and analyses used were Student’s *t*-test with Welch’s correction, Mann–Whitney *U*-test or one-way anova with Newman–Keuls post-test. Differences with *P*<0·05 were considered statistically significant.

## Results and Discussion

*L. major* but not *L. amazonensis* induces 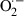 production in CBA macrophage cultures.

The present study aimed to evaluate ROS production by macrophages in response to different stimuli. Uninfected macrophages released very low levels of 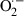 that ranged from 10 to 122 photon counts (*n* = 10) and were similar to those detected in macrophage cultures stimulated with latex beads (38·4–99·10 photon counts) (*n* = 1). By contrast, the positive control cultures stimulated with zymosan particles released high levels of 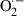 (581·2–7072·2, *n* = 11).

**Figure 1:**
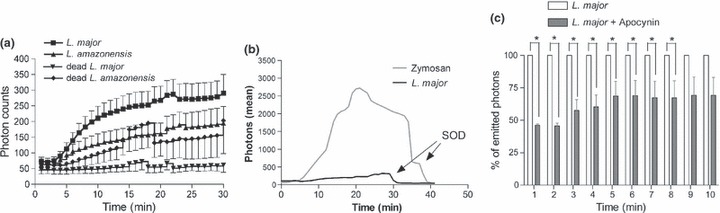
*Leishmania major* promastigotes induce NOX-dependent 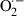 production. Thioglycolate-elicited peritoneal macrophages were incubated with *L.* *major* or *L. amazonensis* promastigotes at a 10:1 ratio at 37°C for 30 min in the presence of lucigenin (25 μm). Control cells were incubated with dead *L.* *major* or dead *L. amazonensis* promastigotes, as well as zymosan, under the same conditions. 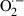 production was measured using lucigenin-based chemiluminescence (CL), expressed in photon counts. *L. major* promastigotes induce the release of significantly higher amounts of 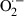 in comparison with *L. amazonensis* (*n* = 11, *P*<0·001, One-way anova and Newman–Keuls), but these levels did not differ significantly from those produced by control macrophage cultures stimulated with dead parasites (a). Lucigenin-based CL decreased in stimulated cell cultures in response to superoxide dismutase (SOD). SOD (2·5 U/mL) was added at the end of each assay, which confirms that photon released in response to *L. major* or zymosan is dependent on 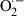 production (one representative experiment out of eight similar experiments) (b). NOX inhibition by apocynin cause partial reduction in lucigenin-based CL. *L. major*-infected cells were pretreated for 18 h with apocynin (500 μm) prior to the addition of parasites. 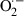 production was detected at 37°C for 10 min and was partially reduced by apocynin. Results are expressed as the percentage of the number of photons emitted by apocynin-treated cells (ranging from 57·6 to 193·9 photons) in relation to untreated macrophages considered as 100% (ranging from 126·2 to 318·7 photons) (*n* = 4, *P*=0·02, Mann–Whitney *U*-test) (c).

Kinetics analysis of 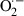 production shows an increase in the 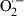 amount when *L. major* promastigotes were added to cells (Figure 1a). By contrast, *L. amazonensis* promastigotes fail to induce the release of significant amounts of 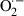 (Figure 1a) which was similar to levels in control nonstimulated macrophages or stimulated with latex beads (data not shown). When dead *L. major* promastigotes were added to macrophage cultures, no increase in photon counts was observed (Figure 1a), which supports the notion that 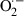 production is dependent on *L. major* viability. The addition of SOD (2·5 UI/mL) at the end of each assay confirms that photon released is as a result of 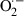 production (Figure 1b).

Next, the participation of NOX assembly in 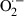 production was evaluated in cells pretreated with apocynin (500 μm). First, pretreatment of *L. major*-infected cells with apocynin was performed and induced a partial reduction on 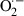 production (*n* = 4, *P* = 0·02, Mann–Whitney; Figure 1c). This partial inhibition of 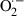 production by apocynin indicates that *L. major*-induced release of 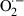 production is dependent on NOX2 and also on a different NOX, such as NOX4. NOX4 is an NADPH-dependent oxidase that is not inhibited by apocynin (20). It is highly expressed in numerous cell types including endothelial cells (22) and embryonic stem cells (23). Although it has been described that NOX4 is involved in other cell functions (24,25), its role in innate immunity has been suggested (26), so it is possible that this oxidase also participates in the 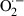 production involved in the control of *Leishmania* infection. Then, *L. amazonensis*-infected macrophages were pretreated with apocynin that did not modify 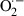 production by these cells (data not shown). This finding suggests that the 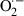 production by CBA macrophages detected during the assay was not dependent upon NOX.

The average value of the maximum number of lucigenin-derived photons released (*R*_max_) by macrophages in response to *L. major* was then calculated and shown to be 276·10 ± 98·08 photon counts, a value 3·5 times higher (*P*<0·05; *n* = 6; Kruskal–Wallis) than the *R*_max_ detected in uninfected macrophage cultures (40·70 ± 10·39 photon counts; *P*>0·05; *n* = 6, Kruskal–Wallis). In addition, the *R*_max_ values of lucigenin-derived photons in macrophage cultures stimulated with *L. amazonensis* (177·30 ± 73·54 photon counts) was not statistically different (*P*>0·05; *n* = 6, Kruskal–Wallis) from those in control macrophages. These findings show that, different from *L. major*, *L. amazonensis* did not trigger 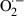 production during phagocytosis.

**Figure 2:**
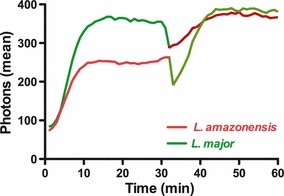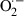 production by macrophages sequentially stimulated with *L. major* or *L. amazonensis* promastigotes. Phagocytic assays were performed by incubating cells with *L. major* (green) or *L. amazonensis* (red) promastigotes for 30 min. Next, the parasite stimuli were switched, and the cells were incubated for a second 30-min period, with either *L. amazonensis* or *L. major* promastigote (10:1), respectively. These sequential stimulations were performed in the presence of lucigenin (25 μm) at 37°C and 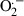 production was measured via lucigenin-based chemiluminescence emitted by cells. *L. amazonensis* did not revert the 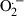 production induced by *L. major* in macrophage cultures, yet incubation with *L.* *major* in cultures previously stimulated with *L. amazonensis* did revert relative low levels of 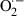 production (one representative experiment out of three similar experiments).

Next, we hypothesized that *L. amazonensis* inhibits 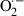 production in response to *L. major* infection. To test this hypothesis, sequential phagocytic assays were then performed by incubating cells with *L. amazonensis* promastigotes for 30 min, followed by a 30-min period of incubation with *L. major*. As expected, macrophages uniquely infected with *L. amazonensis* produced very low levels of 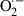 (Figure 2). The addition *of L. major* promastigotes to *L. amazonensis*-stimulated cells reverted the relatively low levels of 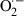 production, which were increased to levels similar to those produced by cells uniquely stimulated with *L. major* (Figure 2). Thereafter, cells were primarily stimulated with *L. major* promastigotes for 30 min, followed by a 30-min period of incubation with *L. amazonensis*. Interestingly, the addition of *L. amazonensis* promastigotes to macrophages previously stimulated with *L. major* did not reverse the *L. major*-induced enhancement of 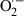 production. 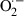 levels remained similar to those produced by macrophages which were exclusively stimulated with *L. major* (Figure 2), showing that *L. amazonensis* promastigotes did not additionally stimulate 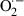 production by macrophages, even when NOX complex was already assembled in response to *L. major* stimulation. In sum, these findings suggest that the events, regarding 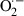 production in response to *L. major* and lack of production in response to *L. amazonensis*, are independent of each other.

The mechanism involved in the failure of 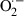 production in *L. amazonensis*-infected cells remained to be elucidated. It is possible that *L. amazonensis* alters ROS production by host cells, using one of the mechanisms that have been previously described for several microbes: (i) *Leishmania donovani* promastigotes delay 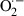 production by preventing NOX assembly and phagosome maturation (5,13,14), subsequent to maintenance of a periphagosomal coat of F-actin (5,13,14,27); (ii) *Leishmania pifanoi* amastigotes avoid 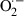 production by inducing an increase in haeme degradation. This results in blockage of the maturation of gp91_phox_ subunit of NOX, and, subsequently, prevents assembly of the NOX complex (15); (iii) *Salmonella typhimurium* reduces 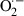 production by removing cytochrome b_558_ subunit from the phagosomal membrane of infected macrophages (16,28); and (iv) *Helicobacter pylori* recruits to nascent phagosomes cytochrome b_558_, yet does not efficiently acquire or retain p47_phox_ or p67_phox_ components of NOX. This results in disruption of NOX, lack of ROS accumulation inside phagosomes and 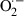 release into the cytoplasm (29).

### *L. major* induces H_2_O_2_ accumulation in macrophage cultures

**Figure 3:**
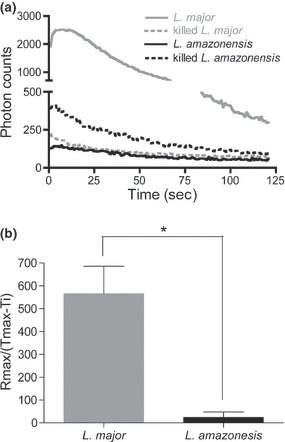
*L. major* promastigotes induce higher levels of H_2_O_2_ accumulation. Thioglycolate-elicited peritoneal macrophages were incubated with *L. amazonensis* or *L. major* promastigotes (10:1) for 30 min at 37°C. H_2_O_2_ accumulation was measured in cell supernatants by chemiluminescence decay in the presence of luminol (25 μm) and microperoxidase (80 nm) for an additional two min. The highest amount of H_2_O_2_ was detected in supernatants collected from live *L.* *major*-stimulated macrophages (one representative experiment out of five similar experiments) (a). Differences in H_2_O_2_ accumulation between *L. major*- and *L. amazonensis*-infected cells are expressed as the maximal oxidative responses for a given time interval [*R*] (as described in Materials and Methods). *L. major*-infected macrophages accumulated twenty times more H_2_O_2_ than *L.* *amazonensis*-infected cells (*n* = 3, *P*=0·04, Student’s *t-*test with Welch’s correction) (b).

ROS generation is a process involving a cascade of events that begins with 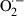 production, which dismutates into H_2_O_2_ either spontaneously, especially at low pH levels, or via a mechanism dependent on SOD (30,31). *In vitro* experiments demonstrated a dose-dependent leishmanicidal effect of H_2_O_2_ against *L. donovani*, *Leishmania tropica* and *Leishmania chagasi* promastigotes (11). Using the phenol red method, we described previously that *L. amazonensis* induced the accumulation of half as much H_2_O_2_ as was accumulated in *L. major*-infected macrophages (17). To confirm this, we measured peroxide levels using the more sensitive luminol-based CL method to determine microperoxidase-induced decay of H_2_O_2_ (19). The highest amount of H_2_O_2_ was detected in supernatants from live *L. major*-infected macrophages (Figure 3). To illustrate the differences in H_2_O_2_ accumulation between *L. major*- and *L. amazonensis*-infected cells, the maximal oxidative responses for a specific time interval were calculated using the equation *R*= *R*_max_/(*T*_max_ − *T*_*i*_) (21). Figure 3(b) illustrates the *R* values corresponding to H_2_O_2_ accumulation in supernatants of *L. major*- or *L. amazonensis*-stimulated macrophages. These findings reveal that H_2_O_2_ accumulation in supernatants of *L. major*-stimulated macrophages was 20 times greater than in *L. amazonensis*-stimulated cells (*P*=0·04, Student’s *t*-test with Welch’s correction; Figure 3b).

The luminol-microperoxidase method was used to distinguish H_2_O_2_ accumulation from the production of others ROS, which are also detected by luminol-based CL, such as 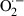 (32). In addition to the highest levels of 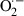 produced by *L. major*-infected macrophages, it is also conceivable that the initial cellular production of 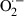, followed by subsequent dismutation into H_2_O_2_, was likely responsible for the elevated levels of ROS detected in supernatants (Figure 3). The data presented herein do not rule out the possibility that other ROS besides H_2_O_2_ are released in *L. major*-infected cultures. In fact, there is evidence that inside macrophages, H_2_O_2_ can be converted in a variety of other ROS, such as ^•^OH, HOCl^−^ (33) and ONOO^−^. As ONOO^−^ exhibited a great toxic effect against *Leishmania*, it is possible that this compound plays a crucial role in *L. major* killing inside macrophages from CBA mice (6). Furthermore, the cellular and molecular mechanisms whereby ROS exert their cytotoxic activities are not yet fully described for *Leishmania* (11). Regarding 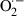 and H_2_O_2_ microbicidal activity (30,34), we suggest that these molecules may contribute to intracellular events resulting in *L. major* killing inside CBA macrophages.

## Concluding Remarks

Previous study using the phenol red method showed that *L. amazonensis* induced the accumulation of half as much H_2_O_2_ as was accumulated in *L. major*-infected inflammatory macrophages (17). These data are in accordance with this study which employed comparative and real-time CL assay, a high-sensitive approach that evaluates ROS production in cell cultures (35,36). A recent study has demonstrated that *Leishmania mexicana*, a parasite species closely related to *L. amazonensis*, diminished ROS production in PMA-stimulated macrophages from both BALB/c and C57BL/6 mice (37). Nonetheless, this study is the first report, which demonstrates that two distinct species of *Leishmania* markedly triggered the production of different levels of ROS in macrophages from a unique mouse strain. The fact that *L. amazonensis*-infected cells release lower amounts of ROS, in comparison with either uninfected or *L. major*-infected macrophages, suggests that the inability of CBA macrophages to destroy *L amazonensis* parasites (7) depend, at least partially, on inefficient ROS production. It has been recently demonstrated by Khouri *et al.* (12) that exposition of *Leishmania braziliensis*- or *L. amazonensis*-infected cells to increasing levels of 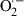 induced a severe reduction in the number of intracellular parasites, demonstrating an effective role for 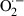 in intracellular parasite killing. Other authors have shown that a low ROS production by *Leishmania*-infected macrophages is a result of the parasite antioxidative response for ROS production (38,39). However, we present evidence against this idea, because *L. major* and *L. amazonensis* parasites did not exhibit any 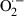 production and H_2_O_2_ formation when incubated alone with lucigenin or luminol, respectively (data not shown) and also did not exhibit any anti-oxidative responses when incubated with 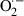 and H_2_O_2_ donors (data not shown). Alternatively, the inability of CBA macrophages to kill *L. amazonensis* may depend on interactions between parasite surface molecules and macrophage receptors (38–40), which may lead to the modulation of host-cell signalling pathways (41) and a macrophage deficiency in the activation of parasite innate killing mechanisms (42). Also viable parasites can express different surface molecules able to interact with macrophage’s surface receptors necessary to induce ROS production. We showed that the genetic background of the host determines the relative degree in which the parasite could be modulating the oxidative response, but further experiments need to be performed to determine the exact mechanism involved in the impairment of ROS production in *L. amazonensis*-infected CBA macrophages.
